# Meckel’s Diverticulum Mimicking a Postoperative Flange with Acute Intestinal Obstruction and Midgut Volvulus: A Case Report

**DOI:** 10.70352/scrj.cr.24-0079

**Published:** 2025-02-06

**Authors:** Stéphane Kohpe Kapseu, Venant Tchokonte-Nana

**Affiliations:** 1 Cliniques Universitaires des Montagnes, Bangangté, West, Cameroon; 2 Faculty of Health Sciences, Comparative Anatomy and Experimental Histopathology and Surgery, Université des Montagnes, Bangangté, West, Cameroon

**Keywords:** Meckel’s diverticulum, midgut volvulus, malrotation, appendicitis, intestinal obstruction, case report

## Abstract

**INTRODUCTION:**

The interest of this case lies in the exceptional and rare character of the observed association.: Meckel’s diverticulum (MD) mimicking a postoperative flange complicated by acute intestinal obstruction and malrotation by midgut volvulus.

**CASE PRESENTATION:**

A 17-year-old black male student, with a body mass index of 28 kg/m^2^ was admitted to the emergency department of a 4th category rural hospital, with paroxystic abdominal pain and vomiting. Medical history revealed an abdominal surgery for an umbilical hernia 3 years earlier. There was no malformation such as imperforate anus, Hirschsprung's disease, esophageal tracheal fistula, or cardiac anomaly in the medical history. An abdominal X-ray confirmed an acute intestinal obstruction showing hydroaeric levels. The diagnosis of acute intestinal obstruction on a flange was retained. A median laparotomy was performed; a solid mass-like lengthy structure mimicking postoperative flange was seen associated with midgut volvulus, while a malposition of the intestine was observed with a mesenteric band, as well as a hyperemic appendix. A 90° rotation stop of the midgut also called a complete common mesentery was in place; we then carried out a Ladd procedure. Morpho-pathological examination of the surgical specimens revealed the following: richly vascularized fibro-adipose tissues with no evidence of malignancy in the diverticular specimen, and acute pan-appendicitis with no evidence of malignancy in the appendicular specimen. The patient started to ingest food orally on the third postoperative day, and he was discharged uneventfully on the fifth day.

**CONCLUSION:**

MD, although generally a tubular structure, may sometimes appear as a non-tubular mass during clinical examination. Intestinal obstruction due to MD associated with midgut volvulus is exceptional. Management of this association should be based on accurate knowledge of the morpho-embryological specificities during gut development.

## Abbreviations

IM
: intestinal malrotation
MD
: Meckel’s diverticulum

## INTRODUCTION

Meckel’s diverticulum (MD) is found in roughly 2% of the population while intestinal malrotation (IM) is a rare congenital abnormality occurring in 0.2%–1% of the population.^1,2)^ These congenital anomalies (MD and IM) have been recently subjects of literature reviews.^3,4)^ However, internal hernias and midgut volvulus are two rare entities.^2,5,6)^ The interest of this case lies in the exceptional and rare character of the observed association: MD mimicking a postoperative flange complicated by acute intestinal obstruction, and IM by midgut volvulus. We present the case of a male adolescent with a history of umbilical hernia repair, operated on in a 4th category rural hospital. Intraoperatively, an unusual association of MD was discovered, complicated by acute intestinal obstruction, IM by midgut volvulus, and acute appendicitis.

## CASE PRESENTATION

A 17-year-old black male student, with a body mass index of 28 kg/m^2^ was admitted to the emergency department of a 4^th^ category rural hospital, with paroxystic abdominal pain and vomiting. Seventy-two hours earlier, he had complained of sudden, spontaneous abdominal pain, postprandial vomiting, as well as the cessation of feces and gas.

On admission, all parameters were good except for a decrease in arterial pressure, and there was no fever. Physical examination revealed a short surgical scar in the umbilical region, with a soft and painful abdomen to palpation. There were no peristaltic sounds on abdominal auscultation while the rectal examination was normal. Medical history revealed an abdominal surgery for an umbilical hernia 3 years earlier. There was no malformation such as imperforate anus, Hirschsprung’s disease, esophageal tracheal fistula, or cardiac anomaly in the medical history.

**Fig. 1:**
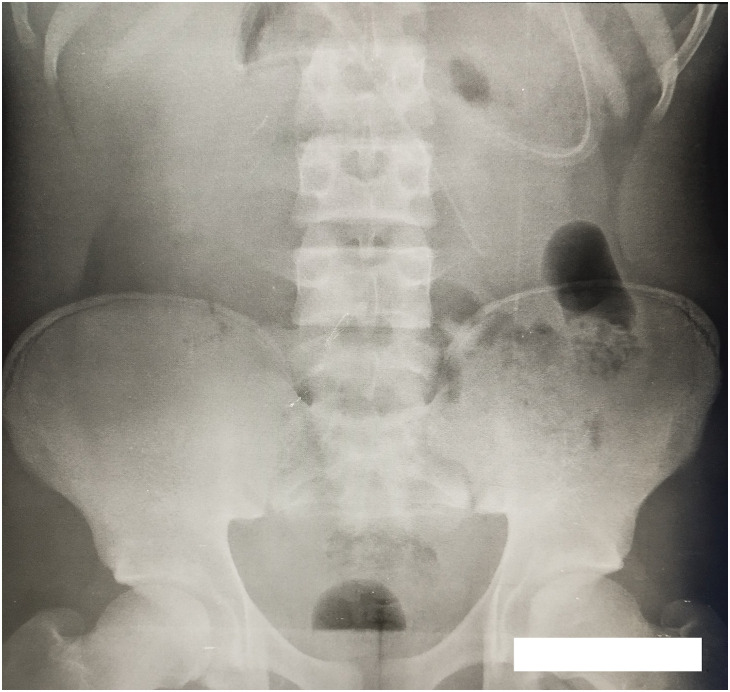
Plain abdominal X-ray showing hydroaeric levels.

The blood count showed no neutrophilic hyperleukocytosis, C-reactive protein was normal, and the sedimentation rate was 50 mm at the first hour and 115 mm at the second. An abdominal X-ray confirmed an acute intestinal obstruction showing hydroaeric levels (**Fig. 1**). No other imaging tests were performed. The diagnosis of acute intestinal obstruction on a flange was retained.

Prior to emergency laparotomy, treatment consisted of peripheral IV rehydration, injectable antispasmodic and antalgic, nasogastric intubation, and Foley catheter to assess diuresis.

**Fig. 2:**
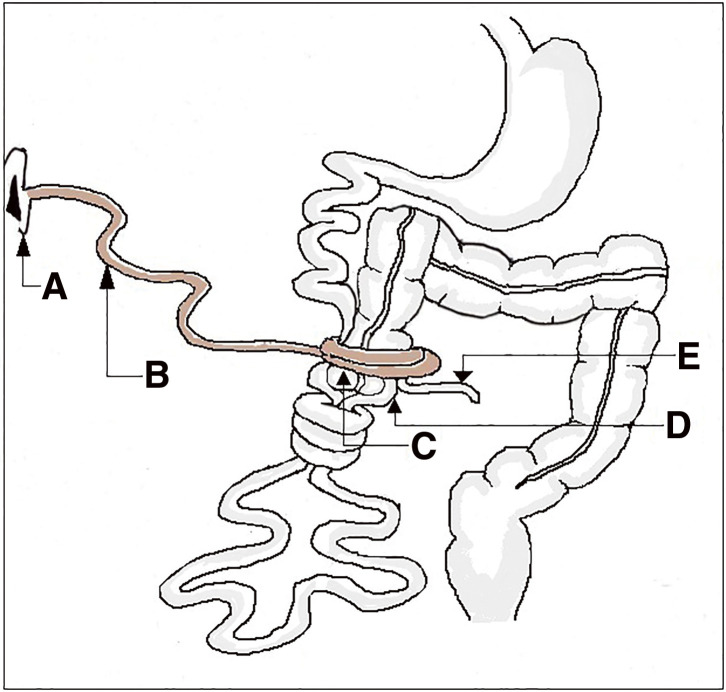
Schematic diagram of intraoperative findings. (**A**) Umbilicus, (**B**) MD, (**C**) cecum, (**D**) ilium, and (**E**) appendix. MD, Meckel’s diverticulum

**Fig. 3:**
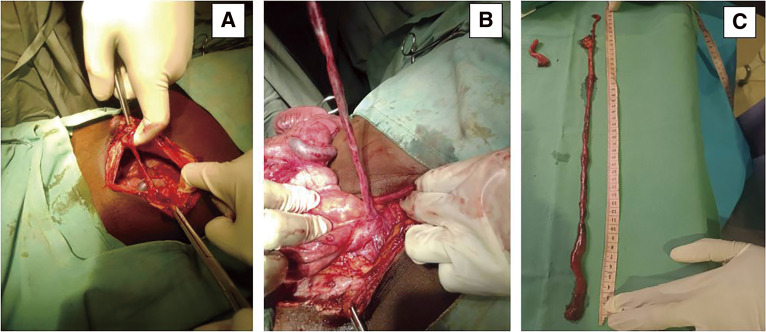
The intraperitoneal view shows (**A**) the retro umbilical origin of MD, (**B**) the transversal colic insertion of MD, and (**C**) the appendix and MD after removal. MD, Meckel’s diverticulum

**Fig. 4:**
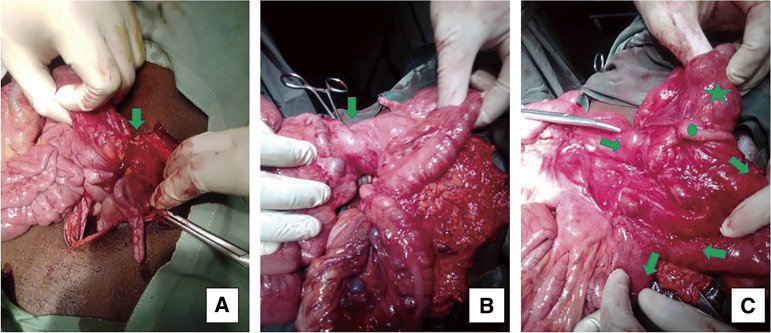
The abdominal content out of its cavity: the green arrow (**A**) indicates the constriction zone of MD, (**B**) the spire of midgut immediately under the cecum, and (**C**) the disposition of midgut after disincarceration and adhesiolysis. (Green circle: appendix; Green star: cecum).

A median laparotomy was performed; there was no free abdominal fluid on exploration, but a solid mass-like lengthy structure mimicking postoperative flange was seen associated with midgut volvulus, while a malposition of the intestine was observed with a mesenteric band, as well as a hyperemic appendix. In the umbilical region, a diverticulum was observed arising from the posterior surface of the umbilicus. This diverticulum exhibits multiple tortuous turns around the midgut and envelops the appendix, leading to a stricture of the appendix (**Figs. 2****–****4**). The cecum and appendix were mobilized, and a division of the mesenteric bands was performed, revealing the duodenojejunal angle and the proximal midgut located in the right lower quadrant of the abdomen, without attachment to the retroperitoneum and with a narrow mesenteric base, while the ascending colon was displaced to the left (**Fig. 2**). A volvulus was observed around the cecum, encircling the ileocecal segment of the midgut (**Figs. 2** and **4**). The uncoiled MD had a fibrous termination on the ileal portion of the intestine (**Fig. 3**). A 90° rotation stop also known as complete common mesentery was characteristic of the midgut malrotation (**Table 1**; type 1).

**Table 1:** Classification of different types of intestinal malrotation by Ribeiro et al.^2)^

Type of intestinal rotation	Description	Details
Type 1	Non-rotation	The entire small intestine, including the duodeno-jejunal angle, is on the right of the spine, and the entire colon is on the left.
Type 2	Reverse rotation	the duodenojejunal angle lies in front of the transverse colon
Type 3	Incomplete rotation	The ileocecal junction comes to rest in the sub-hepatic region.
Type 4	Incomplete rotation	Ladd’s bands are only on the transverse colon.

The treatment of MD typically involves resection of the segment of the midgut containing the diverticulum, followed by a termino-terminal anastomosis. In our case, however, the MD was excised without intestinal resection; indeed, it was a fibrous attachment and was not patent and continuous with the lumen of the midgut. So, a ligature was made at a section of the attachment. A Ladd procedure was then carried out: the volvulus (**Fig. 2**) was untwisted by rotating the bowel in a counterclockwise direction while mesenteric bands were divided before carrying out an appendectomy. The viability of the intestine was good. and the small bowel was placed on the right abdomen, while the large bowel on the left of the abdomen.

The postoperative flange-like diverticulum measured about 45 cm long (**Fig. 3**). The morpho-pathological examination of the surgical specimens revealed the following: richly vascularized fibro-adipose tissues with no evidence of malignancy, no inflammation nor ectopic tissues in the diverticular specimen of approximately 0.5 cm caliber, and acute pan-appendicitis—involvement of all layers of the appendix, with no evidence of malignancy in the appendicular specimen of approximately 0.4 cm caliber.

The postoperative course was uneventful. The nasogastric tube was removed on the third postoperative day after transit had returned. The patient started to ingest food orally on the third postoperative day and was discharged uneventfully on the fifth day. There was no complaint at the first-month follow-up.

## DISCUSSION

An incomplete canalization of the vitelline duct (also known as the omphalomesenteric duct) can be observed during development.^7)^ Normally, this duct obliterates and disappears as the embryo develops.^8,9)^ However, if the duct does not completely canalize, it can lead to various anomalies such as MD, vitelline cyst, and vitelline fistula.^7–9)^ IM results from abnormal fixation and rotation of the gut tube during fetal development.^4,5)^ Preoperative diagnosis of complicated MD and IM is difficult as it mimics other common acute abdominal conditions; the clinical presentation can often be atypical.^3,10)^ Indeed, these two congenital malformations are generally discovered intraoperatively.^3,5,11)^ In this case, classic obstructive syndrome presents a diagnostic and therapeutic challenge, especially for having MD, midgut volvulus, and acute appendicitis. Usually, symptoms include persistent nausea, biliary vomiting, abdominal distension, absolute constipation, and sometimes painless gastrointestinal bleeding.^3)^ The patient reported early postprandial vomiting and abdominal pain; his abdomen was not distended and was soft, favoring in general a small bowel obstruction. These events are characteristic of IM, a congenital condition caused by insufficient normal intestinal rotation following a physiologic gut herniation.^1)^ According to the classification set out by Ribeiro et al.,^2)^ a 90° rotation stop known as a complete common mesentery was characteristic of this case (**Table 1**; type 1).

Small bowel volvulus can be categorized either as primary (no obvious predisposing factors) or as secondary (specific predisposing anatomical abnormalities). However, numerous etiological factors have been suggested to contribute to the process of primary small bowel volvulus, while adhesion bands, pregnancy, jejunal diverticulum, and/or complications following laparoscopic cholecystectomy are the underlying causes of the secondary type.^12)^ From the latter, midgut volvulus in this case can be categorized as secondary.

An MD occurs consequent to incomplete obliteration of the vitelline or omphalomesenteric duct, which connects the developing intestines to the yolk sac.^3)^ However, MD may appear as a non-tubular mass.^13)^ Although it is generally a tubular or pouch-like structure, it can sometimes be seen on imaging or during clinical examination as a mass or lump in the abdominal region, particularly between the umbilicus and the ileocecal valve.^14–16)^ This longitudinal mass may appear non-tubular because the diverticulum may not be completely filled or dilated, or because of inflammation or other local changes,^13,15)^ making the MD look like another structure or a postoperative abnormality, even in the absence of previous surgery.^17–19)^ Similarly, this case presented MD’s lumen filled with a richly vascularized fibro-adipose tissue with no sign of malignancy mimicking a postoperative flange, confirming the fact that MD may be misdiagnosed as a postoperative flange.^10,11)^

Imaging tests such as abdominal computed tomography (CT) and magnetic resonance imager (MRI) recommended in the literature^1,3,4,10,11)^ were not available in our facility, only an abdominal X-ray was performed on the patient.

The history of abdominal surgery and the fibroelastic nature of MD led to the initial suggestion of a postoperative flange.

Although several reported cases have resulted in intestinal resection,^3,10,11)^ there was an absence of communication between the lumen of the midgut and the MD, which led us not to perform a bowel resection. Indeed, the postoperative flange-like MD was resected between two forceps. In general, late correction of malrotation is not recommended,^4)^ so, in this case, the small intestine was just repositioned in the right iliac fossa and the colon on the left.

## CONCLUSION

Intestinal obstruction due to non-patent MD associated with midgut volvulus is exceptional. Management of this association is based on accurate knowledge of the morpho-embryological specificities during gut development.

## ACKNOWLEDGMENTS

The authors wish to thank all the medical team at the Cliniques Universitaires des Montagnes.

## DECLARATIONS

### Funding

This study was not funded.

### Authors’ contributions

All authors have read and approved the manuscript, and they are responsible for the manuscript.

### Availability of data and materials

Not applicable.

### Ethics approval and consent to participate

This work does not require ethical considerations or approval.

### Consent for publication

Written informed consent was obtained from the patient and for any accompanying images.

### Competing interest

Authors have no competing interests.
